# Reassessment of *Varroniabellonis* - a threatened, endemic plant from Puerto Rico

**DOI:** 10.3897/BDJ.9.e64654

**Published:** 2021-04-01

**Authors:** Martin A. Hamilton, Anthony Maldonado-Castro, José A. Sustache, Omar A. Monsegur-Rivera

**Affiliations:** 1 Royal Botanic Gardens, Kew, London, United Kingdom Royal Botanic Gardens, Kew London United Kingdom; 2 University of Puerto Rico at Rio Piedras, San Juan, Puerto Rico University of Puerto Rico at Rio Piedras San Juan Puerto Rico; 3 Puerto Rico Department of Natural and Environmental Resources, San Juan, Puerto Rico Puerto Rico Department of Natural and Environmental Resources San Juan Puerto Rico; 4 US Fish and Wildlife Service, Cabo Rojo, Puerto Rico US Fish and Wildlife Service Cabo Rojo Puerto Rico

**Keywords:** Boraginaceae, Caribbean flora, conservation, endangered species, endemism, Red List

## Abstract

**Background:**

*Varroniabellonis* (Urb.) Britton is a lianescent or recumbent shrub that is endemic to Puerto Rico where it is restricted to specific geology types with a limited extent on the western half of the Island. The species occurs on serpentinite geology covered by serpentine-derived soils in the west-central mountains and on limestone geology in the the northern karst region. The species area of occupancy is estimated to range between 108 km^2^ and 268 km^2^ and its extent of occurrence to be between 644 km^2^ and 852 km^2^. The number of locations are estimated to be four. There are 418 known mature individuals in the wild (Hamilton 2020a). The species was previously assessed as Critically Endangered (Linsky and Sustache 2014), based on available information. However, an international team have been collaborating to conserve the species and, based on new information derived from this work, the species is reassessed as Endangered (EN), based on Criteria B1ab(i,ii,iii,iv,v)+2ab(i,ii,iii,iv,v), according to the IUCN Red List Categories and Criteria (version 3.1) and guidelines (IUCN Standards and Petitions Committee 2019).

**New information:**

Areas of suitable habitat across the native range of the threatened plant, *V.bellonis*, were surveyed by a team of experts between 2016 and 2019 to determine the species habitat preferences, identify threats to the species survival and provide an up-to-date meta-population status. The new information enabled members of the international team to reassess the species status and will enable sound and scientifically-based recovery actions to be recommended that can secure *Varroniabellonis* populations for the future. Parallel efforts are ongoing to explore the species population genetics and reproductive biology.

## Introduction

In this paper, we present a species conservation profile for a species endemic to the Commonwealth of Puerto Rico.

## Species Conservation Profiles

### Varronia bellonis

#### Species information

Scientific name: Varroniabellonis

Species authority: (Urb.) Britton

Synonyms: Cordiabellonis Urb.

Common names: Unknown

Kingdom: Plantae

Phylum: Tracheophyta

Class: Lamiids

Order: Boraginales

Family: Boraginaceae

Taxonomic notes: This species was originally described as *Cordiabellonis* by Urban in Symbolae Antillanae, 1: 393. 1899. It was later transferrred to *Varronia* by Britton and Wilson (1925). *Varroniabellonis* is endemic to the Island of Puerto Rico, where three additional regional endemic species occur, *V.lima* Desv., *V.rupicola* (Urb.) Britton and *V.wagnerorum* (R.A. Howard) Borhidi. The boundaries between these taxa are currently clear and there are no known taxonomic issues for these species. However, further research into the biogeography and phylogenetics of this group is ongoing.

Region for assessment: Global

#### Editor & Reviewers

##### Reviewers

Reviewers: Malin Rivers, Steven Bachman

##### Editor

Editor: Martin A. Hamilton

#### Reviewers

Reviewers: Malin Rivers, Steven Bachman

#### Editor

Editor: Martin A. Hamilton

#### Geographic range

Biogeographic realm: Neotropical

Countries: Puerto Rico

**Figure 1.**
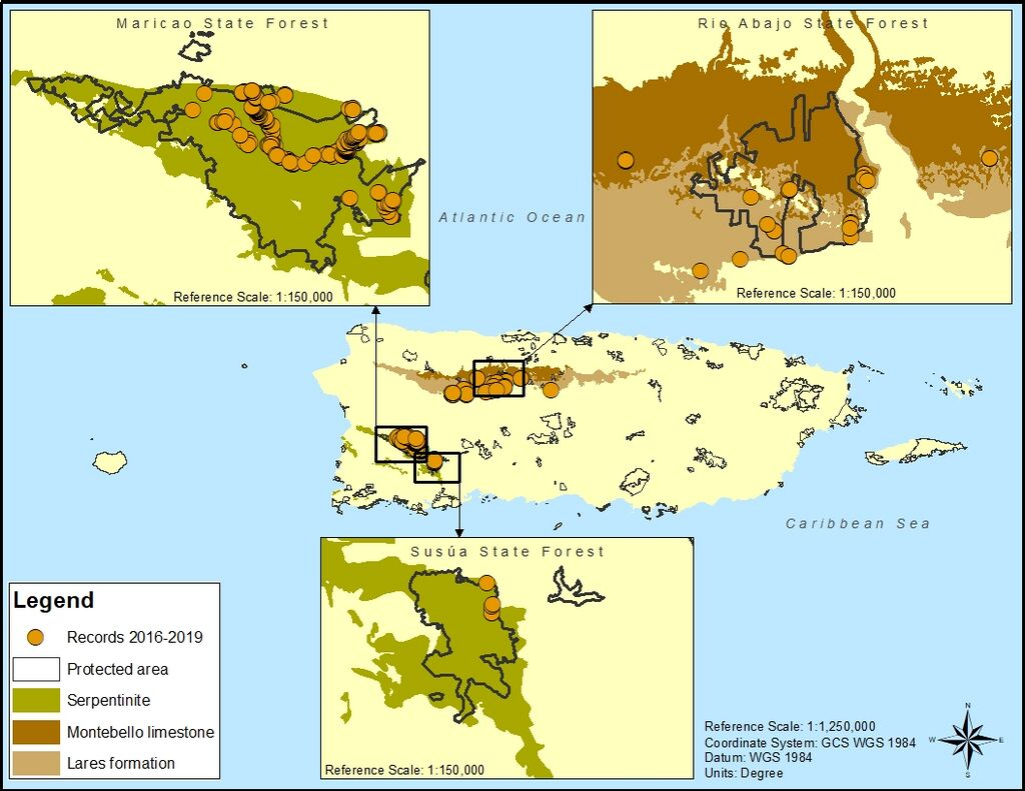
Global distribution of *Varroniabellonis* derived from records collected using GPS between April 2016 and December 2019 showing protected area boundaries across the Commonwealth of Puerto Rico and geology types relevant to the species.

Map of records (image): Fig. 1

Map of records (Google Earth): Suppl. material 1

Basis of EOO and AOO: Observed

Basis (narrative): This species is endemic to Puerto Rico (Acevedo-Rodriguez and Strong 2012) and has a restricted range. Extant individuals were found in and around the boundaries of the Maricao, Susúa and Río Abajo State Forests, the three historical areas of distribution, as well as individuals in previously unrecorded locations in the Municipalities of Arecibo, San German, Sabana Grande, Utuado and Lares.

Min Elevation/Depth (m): 216

Max Elevation/Depth (m): 893

Range description: *Varroniabellonis* was originally described from 1884 collections by Paul Sintenis, number 310, from Indiera Fría Ward and number 310b from Monte Alegrillo, in the Municipality of Maricao (Urban 1899). Over the next century, the species was sporadically collected near the type localities and in the adjacent Municipality of San Germán. Knowledge of its range expanded in the 1990s when the species was collected by G.J. Breckon, number 4136 from Quebrada Grande in the Municipality of Yauco, in the Susúa State Forest and by F. Axelrod, number 8371 from Río Arriba Ward in the Municipality of Arecibo, in the Río Abajo State Forest. The species was subsequently collected by Axelrod, number 8469 from Cordillera Ward in the Municipality of Ciales and, soon after, in the Municipality of Utuado by A.H. Liogier, number 37379 within the Río Abajo State Forest . Since 2000, the species known range has continued to expand with new collections made across the previously-mentioned Municipalities and several new records in the Municipalities of Sabana Grande (e.g. M.A. Hamilton, number 1788 from Tabonuco Ward) and Lares (e.g. O. Monsegur, number 1730 from Lares Ward).Observation and collection event records made between 2016 and 2019 were considered to calculate minimum values of extent of occurrence (EOO) and area of occupancy (AOO), while maximum values of EOO and AOO were calculated considering all available records and the entire area of suitable habitat covering known geology types that support the species (Maldonado 2019). The EOO was estimated to range between 644 km^2^ and 852 km^2^. The AOO was estimated to range between 108 km^2^ and 268 km^2^. Both calculations for EOO and AOO trigger evaluation under the IUCN Red List criterion B, threatened category of Endangered (IUCN Standards and Petitions Committee 2019) and are based on a 2 x 2 km cell size and calculated with GeoCAT (Bachman et al. 2011). The number of locations was calculated to be four, considering threats posed, which can vary depending on whether individuals are found within or outside protected areas. Suppl. material 1A specimen of *Varroniabellonis*, collected by N.L. Britton (number 4287) in 1915 held at UPR (barcode UPR05414), was erroneously associated with Monte Cerrote near the Municipality of Adjuntas, outside the species recorded geology types, due to the wrong pre-printed label with location information being used for the specimen. The first duplicate, held at New York Botanical Garden (barcode NY00967240), resolved the issue by clarifying the collection location as Maricao, within the species known historic range and suitable habitat.

#### New occurrences

#### Extent of occurrence

EOO (km2): 644-852

Trend: Unknown

Causes ceased?: Unknown

Causes understood?: Unknown

Causes reversible?: Unknown

Extreme fluctuations?: Unknown

#### Area of occupancy

Trend: Unknown

Causes ceased?: Unknown

Causes understood?: Unknown

Causes reversible?: Unknown

Extreme fluctuations?: No

AOO (km2): 108-268

#### Locations

Number of locations: 4

Justification for number of locations: The number of locations was calculated to be four considering threats posed by human disturbance, such as road and infrastructure maintenance and land use at the different sites where the species has been recorded.

Trend: Unknown

Extreme fluctuations?: No

#### Population

Number of individuals: 418

Trend: Increase

Justification for trend: Direct observation of new individuals.

Causes ceased?: Unknown

Causes understood?: Unknown

Causes reversible?: Yes

Extreme fluctuations?: No

Population Information (Narrative): The species was severely impacted by a road development project in the northern karst that resulted in hundreds of individuals being lost (U.S. Fish Wildlife Service 1999). Across the species range, habitat is being impacted by development and maintenance of roads, trails and infrastructure (Hamilton 2017a). Surveys between 2014 and 2019 recorded a total of 418 mature individuals, 117 saplings and 121 seedlings across the species known range (Hamilton 2020a, Hamilton 2020b). These findings contradict work by Sánchez-Cuervo et al. (2014) that indicated populations in and around Rio Abajo State Forest and Susua State Forest were extirpated and populations in and around Maricao State Forest were declining. The large size of many individuals recorded in and around Rio Abajo State Forest (Hamilton 2016) and Susua State Forest (Hamilton 2018) between 2014 and 2019 indicate that the species had not been extirpated from these locations and the discovery of individuals at all stages of development (from seedling through to maturity) in and around Maricao State Forest (Hamilton 2020b) indicate that the species had not been declining at this location.

#### Subpopulations

Trend: Unknown

Extreme fluctuations?: No

Severe fragmentation?: No

#### Habitat

System: Terrestrial

Habitat specialist: Yes

Habitat (narrative): Recent studies by Maldonado (2019) revealed that 86% of individuals were found on serpentinite geology covered by serpentine soils in the west-central mountains between 216 and 893 metres. The remaining 14% of individuals were recorded on limestone geology, within the Montebello limestone and Lares formation limestone, in the northern karst region between 273 and 449 metres. The species is predominantly found in undisturbed forest types with most individuals (69%) recorded in evergreen forest growing on serpentine soils. The serpentinite geology covered by serpentine soils in the west-central mountains and on limestone geology in the western part of the northern karst region have 331 km^2^ and 479 km^2^ of extant preferred land cover types, respectively (Maldonado 2019).

Trend in extent, area or quality?: Decline (inferred)

##### Habitat

Habitat importance: Major Importance

Habitats: 1.9. Forest - Subtropical/Tropical Moist Montane

##### Habitat

Habitat importance: Major Importance

Habitats: 1.6. Forest - Subtropical/Tropical Moist Lowland

##### Habitat

Habitat importance: Marginal

Habitats: 14.6. Artificial/Terrestrial - Subtropical/Tropical Heavily Degraded Former Forest

#### Habitat

Habitat importance: Major Importance

Habitats: 1.9. Forest - Subtropical/Tropical Moist Montane

#### Habitat

Habitat importance: Major Importance

Habitats: 1.6. Forest - Subtropical/Tropical Moist Lowland

#### Habitat

Habitat importance: Marginal

Habitats: 14.6. Artificial/Terrestrial - Subtropical/Tropical Heavily Degraded Former Forest

#### Ecology

Size: Lianescent shrub to 14 m

Generation length (yr): 3

Dependency of single sp?: No

**Figure 2.**
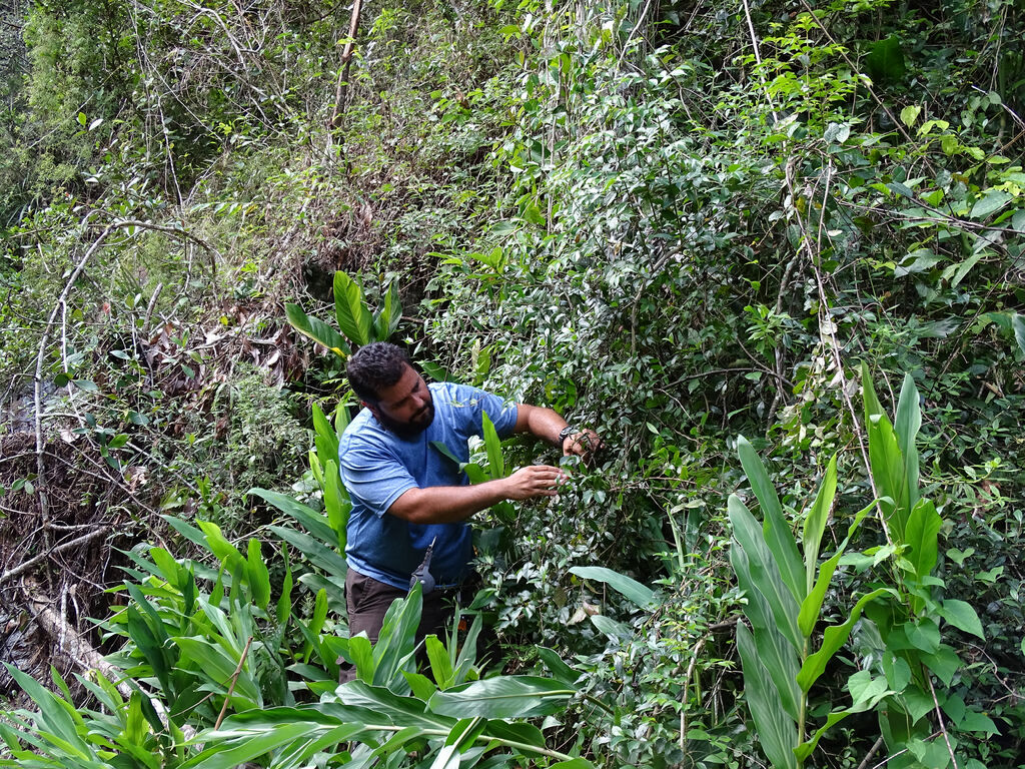
Mr Omar Monsegur examining mature individual of *Varroniabellonis* growing along the bank of a small stream in Maricao Municipality, Puerto Rico. Photo credit: M.A. Hamilton.

**Figure 3.**
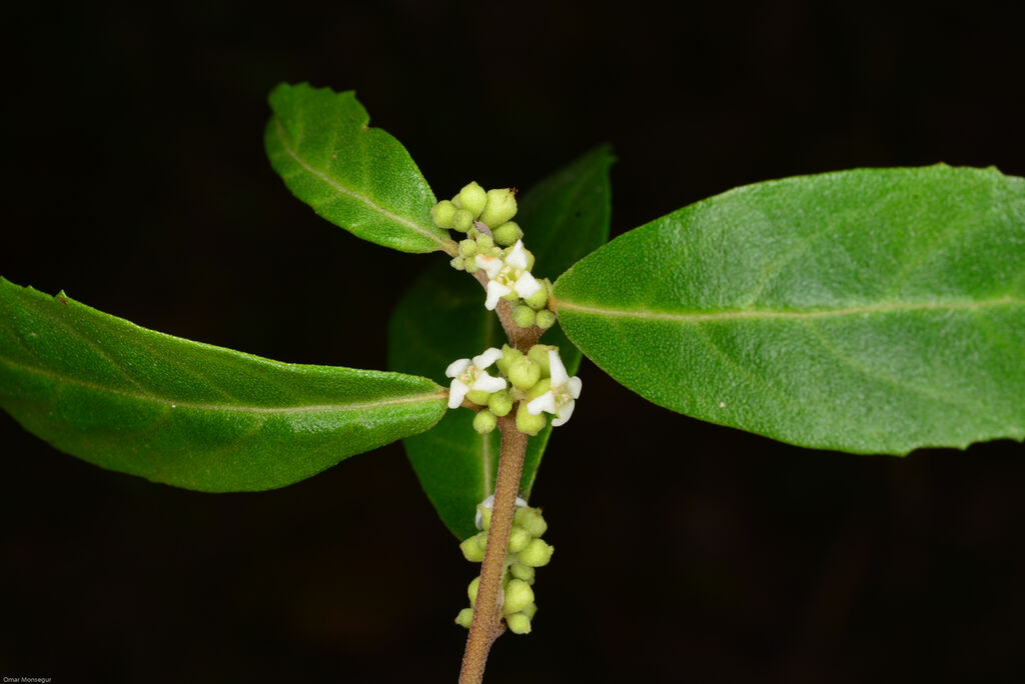
Flowering branch of *Varroniabellonis* collected along the Rio Grande in Sabana Grande Municipality, Puerto Rico. Photo credit: Omar A. Monsegur Rivera.

**Figure 4.**
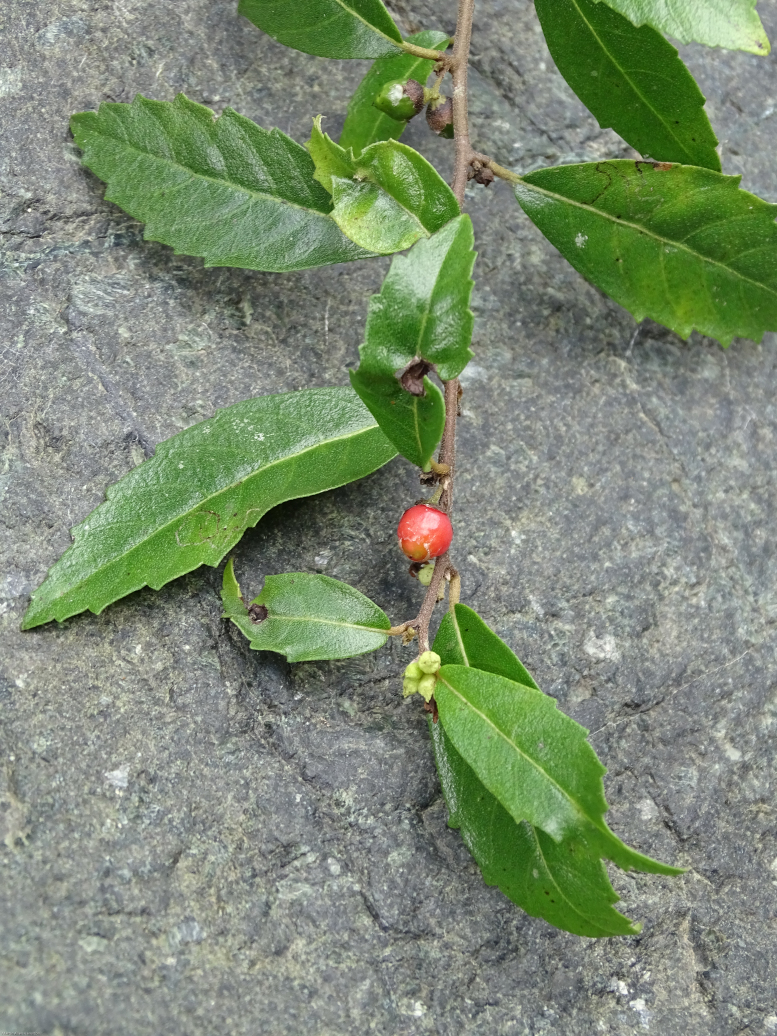
Fruiting branch of *Varroniabellonis* collected along the Rio Grande in Sabana Grande Municipality, Puerto Rico. Photo credit: M.A. Hamilton.

Ecology and traits (narrative): *Varroniabellonis* is a lianescent or recumbent shrub with scandent branches which allow it to clamber into surrounding trees, over surrounding vegetation and across exposed slopes (Fig. 2). In open areas exposed to intense sunlight, the species may remain as a compact, multi-stemmed shrub. Mature individuals have been recorded as clumps over one metre and clambering over fourteen metres in forested areas with dense canopy cover (Hamilton 2017b). The white flowers (Fig. 3) are visited by several insect species, but which species are effective pollinators remains unknown. The fruit is a drupe that turns red when ripe (Fig. 4) and usually develops between November and February. Several bird species have been recorded visiting the plant during the fruiting season and ongoing camera-trapping has shown clear evidence for bird dispersal (Hamilton 2019a). The generation length of this plant is estimated to be 3-5 years; however, research is underway to confirm the species generation length and understand its reproductive biology through field observations and greenhouse studies (Hamilton 2020a, Hamilton et al. 2020).

#### Threats

Justification for threats: When the species was listed as ‘Endangered’ under the United States Endangered Species Act in 2017, the largest population was in Río Abajo State Forest. Unfortunately, much of the population was lost during development of Puerto Rico Highway 10 and efforts to rescue and translocate individuals were unsuccessful (U.S. Fish Wildlife Service 2017). In addition, road maintenance along Highway 120 in Maricao State Forest affected individuals growing in edge habitat along the road (U.S. Fish Wildlife Service 2017). Recent surveys have shown the impacts of drought stress, land use, development and maintenance of infrastructure, pest insects and invasive plant species to be detrimental to the survival of *Varroniabellonis* (Hamilton 2016, Hamilton 2017a, Hamilton 2017b, Hamilton 2019a, Hamilton 2019b, Hamitlon 2019, Hamilton 2020a). These threats impact the species through limitation of recruitment, habitat modification, degradation and/or fragmentation and direct or indirect species mortality. Climate change might already be impacting the species through prolonged periods of drought which are expected to increase in the future, particularly in Susúa State Forest, the northern karst and along the ridges and drier southern slopes of the Maricao Municipality. Droughts may result in higher seedling mortality and a reduced reproductive output due to flower and fruit abortion. Tropical storms have had a direct negative impact on some individuals through mortality; however, the species is a gap coloniser and the majority of individuals responded favourably (e.g. new growth, profuse flowering and fruiting) following Hurricane Maria in 2017 (Hamilton 2018, Hamilton 2019a).

##### Threats

Threat type: Ongoing

Threats: 1.3. Residential & commercial development - Tourism & recreation areas2.1.2. Agriculture & aquaculture - Annual & perennial non-timber crops - Small-holder farming4.1. Transportation & service corridors - Roads & railroads4.2. Transportation & service corridors - Utility & service lines6.1. Human intrusions & disturbance - Recreational activities8.1.2. Invasive and other problematic species, genes & diseases - Invasive non-native/alien species/diseases - Named species

##### Threats

Threat type: Past

Threats: 1.1. Residential & commercial development - Housing & urban areas3.2. Energy production & mining - Mining & quarrying

##### Threats

Threat type: Future

Threats: 11.2. Climate change & severe weather - Droughts

#### Threats

Threat type: Ongoing

Threats: 1.3. Residential & commercial development - Tourism & recreation areas2.1.2. Agriculture & aquaculture - Annual & perennial non-timber crops - Small-holder farming4.1. Transportation & service corridors - Roads & railroads4.2. Transportation & service corridors - Utility & service lines6.1. Human intrusions & disturbance - Recreational activities8.1.2. Invasive and other problematic species, genes & diseases - Invasive non-native/alien species/diseases - Named species

#### Threats

Threat type: Past

Threats: 1.1. Residential & commercial development - Housing & urban areas3.2. Energy production & mining - Mining & quarrying

#### Threats

Threat type: Future

Threats: 11.2. Climate change & severe weather - Droughts

#### Conservation

**Figure 5.**
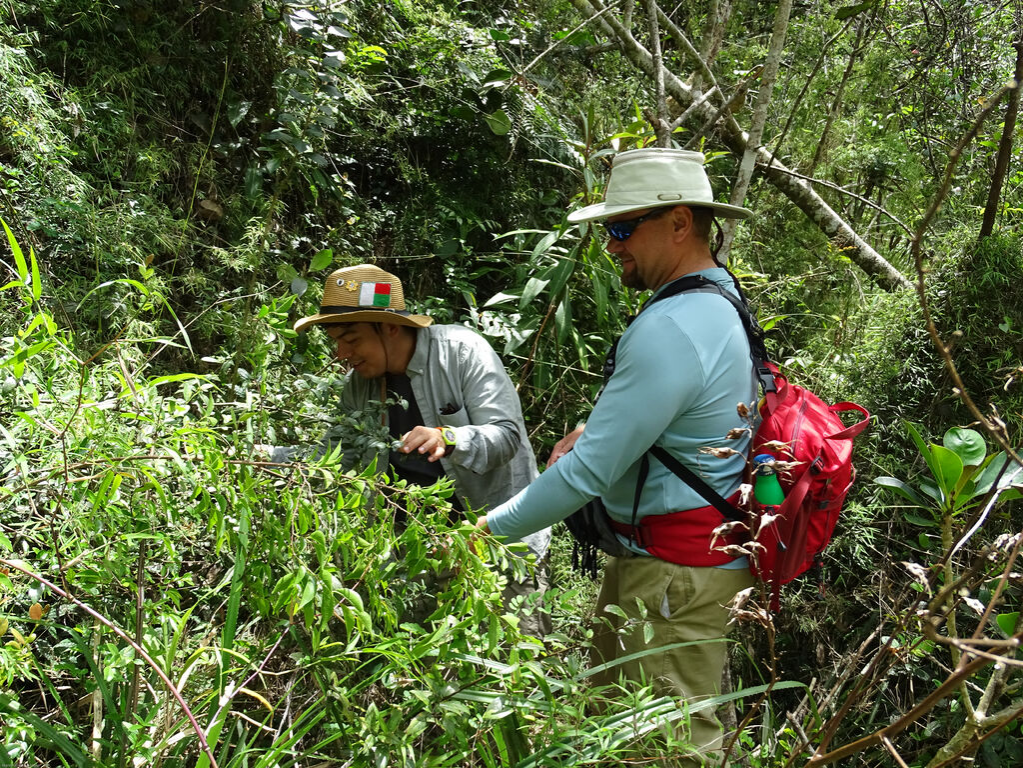
Mr Anthony Maldonado-Castro and Dr Martin A. Hamilton monitoring a mature individual of *Varroniabellonis* growing in Maricao State Forest, Puerto Rico. Photo credit: M.A. Hamilton.

**Figure 6.**
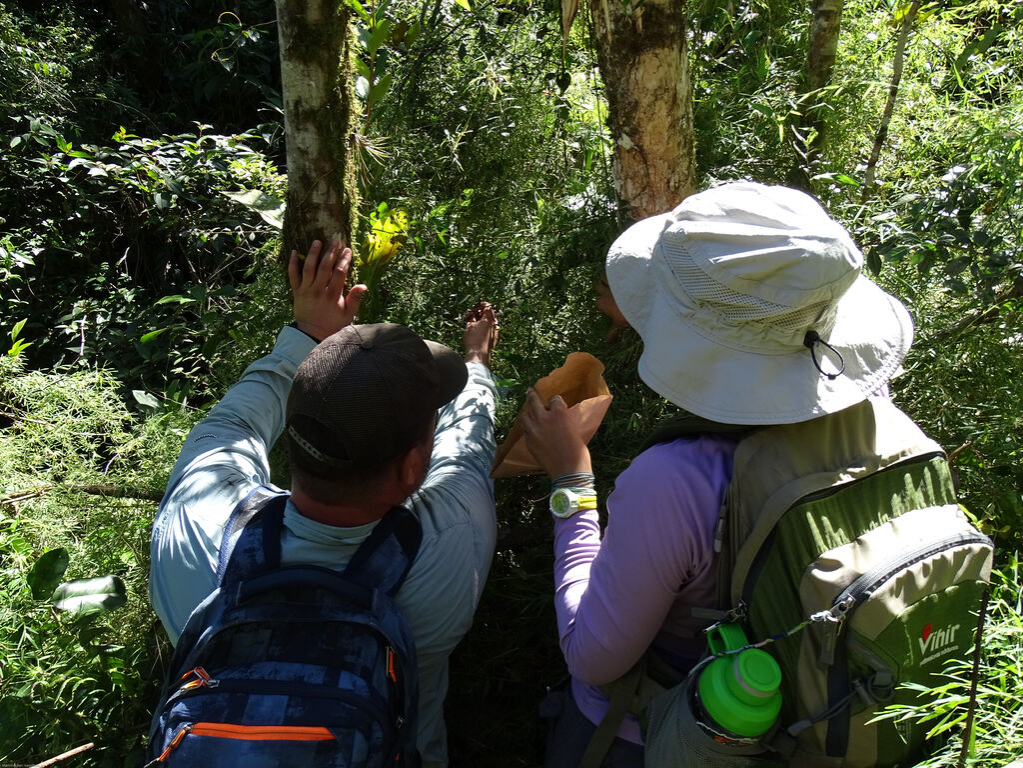
Mr Jesús M. Ríos Cruz and Ms Nahíra Arocho Hernández collecting fruit from a mature individual of *Varroniabellonis* growing along the bank of a small stream in Maricao Municipality, Puerto Rico. Photo credit: M.A. Hamilton.

Justification for conservation actions: There is 186 km^2^ of extant preferred land cover types that support the species within three protected areas (Maldonado 2019). On the limestone geology in the western part of the northern karst region, the species is extant in the Río Abajo State Forest. In the serpentinite geology covered by serpentine soils in the west-central mountains, the species is found in Maricao and Susúa State Forests (Hamilton 2019a, Hamilton 2020a, Hamilton 2020b). This species is listed as ‘Endangered’ under the United States Endangered Species Act as *Cordiabellonis* (U.S. Fish and Wildlife Service 1997) and a recovery plan was approved in 1999 (U.S. Fish Wildlife Service 1999). Hundreds of individuals in the footprint of the Puerto Rico Highway 10 development were transplanted to the Cambalache State Forest nursery and cuttings were taken for asexual propagation. The fate of most of this material is unclear; however, work by Sánchez-Cuervo et al. (2014) suggested material re-introduced at Río Abajo did not survive. A single tagged individual in Río Abajo State Forest, located during recent surveys, is apparently the sole survivor of a transplanting activity from material grown at the Cambalache State Forest nursery (Hamilton 2016). Many individuals have been recorded outside of the existing protected area network requiring close collaboration with land owners to develop management plans and enable *ex-situ* collections of this species to be secured. Collaboration amongst international partners (the Royal Botanic Gardens, Kew; the University of Puerto Rico, Mayagüez Campus; The Puerto Rico Department of Natural and Environmental Resources; and the US Fish and Wildlife Service) has secured *ex-situ* collections of this species at the University of Puerto Rico, Mayagüez Campus nursery and seed bank (Hamilton 2019b, Hamitlon 2019, Hamilton 2020a). The partners monitor individuals (Fig. 5) across the species known range and collect seeds (Fig. 6) when available for recovery purposes. Further development of *ex-situ* collections, including germination and cultivation trials for living plants, is underway to aid the species recovery (Hamilton 2019b, Hamilton 2020a, Hamilton et al. 2020). Ongoing work on the species population genetics, germination requirements and reproductive biology will guide future recovery actions, including augmenting existing populations and further re-introduction efforts.

##### Conservation actions

Conservation action type: Needed

Conservation actions: 1.1. Land/water protection - Site/area protection2.1. Land/water management - Site/area management3.3.1. Species management - Species re-introduction - Reintroduction3.4.1. Species management - Ex-situ conservation - Captive breeding/artificial propagation3.4.2. Species management - Ex-situ conservation - Genome resource bank5.4.2. Law & policy - Compliance and enforcement - National level4.3. Education & awareness - Awareness & communications

##### Conservation actions

Conservation action type: In Place

Conservation actions: 1.1. Land/water protection - Site/area protection3.2. Species management - Species recovery3.4. Species management - Ex-situ conservation5.1. Law & policy - Legislation

#### Conservation actions

Conservation action type: Needed

Conservation actions: 1.1. Land/water protection - Site/area protection2.1. Land/water management - Site/area management3.3.1. Species management - Species re-introduction - Reintroduction3.4.1. Species management - Ex-situ conservation - Captive breeding/artificial propagation3.4.2. Species management - Ex-situ conservation - Genome resource bank5.4.2. Law & policy - Compliance and enforcement - National level4.3. Education & awareness - Awareness & communications

#### Conservation actions

Conservation action type: In Place

Conservation actions: 1.1. Land/water protection - Site/area protection3.2. Species management - Species recovery3.4. Species management - Ex-situ conservation5.1. Law & policy - Legislation

#### Other

##### Use and trade

##### Ecosystem services

#### Use and trade

#### Ecosystem services

#### Viability analysis

## Supplementary Material

1C9A0B9E-1148-5CEA-8774-FC6EC78C3F1F 10.3897/BDJ.9.e64654.suppl1 Supplementary material 1 Varroniabellonis occurrence records from Puerto Rico Data type occurrences map File: oo_398470.kml https://binary.pensoft.net/file/398470 Hamilton, M. A. & Maldonado, A.
